# MRI-based structural covariance network in early human brain development

**DOI:** 10.3389/fnins.2023.1302069

**Published:** 2023-11-01

**Authors:** Dingna Duan, Dong Wen

**Affiliations:** ^1^State Key Laboratory of Cognitive Neuroscience and Learning, Beijing Normal University, Beijing, China; ^2^Beijing Key Laboratory of Brain Imaging and Connectomics, Beijing Normal University, Beijing, China; ^3^IDG/McGovern Institute for Brain Research, Beijing Normal University, Beijing, China; ^4^School of Intelligence Science and Technology, University of Science and Technology Beijing, Beijing, China

**Keywords:** MRI, cortical morphology, structural covariance network, human brain, early development

## 1. Introduction

During the first years of life, the human brain undergoes rapid structural and functional development, laying the foundation for cognitive behavior's progression throughout the individual's life. The structural covariance network (SCN), constructed based on varying cortical morphological features extracted from structural MRI, reflects the coordinated brain maturation between cortical regions (Alexander-Bloch et al., 2013; Gilmore et al., 2018). It is associated with white matter networks, functional networks, cognitive functions, and genetic heritability, making it valuable for early brain development studies (Chen et al., 2011; Gong et al., 2012; Alexander-Bloch et al., 2013; Seidlitz et al., 2018; Fenchel et al., 2020). Regarding the construction of SCN, the most representative method is to estimate the similarity between cortical regions by calculating the correlation of their morphological features [such as cortical thickness (CT), surface area (SA), gray matter volume (GMV), sulcal depth (SD), etc.] at the population level (He et al., 2007; Alexander-Bloch et al., 2013; Gilmore et al., 2018), which has been widely applied in adult and adolescent populations. In recent years, some studies have proposed SCNs constructed at the individual level (Kong et al., 2015; Meng et al., 2015; Li et al., 2017, 2021; Seidlitz et al., 2018; Yu et al., 2018; Sebenius et al., 2023), which can capture individual difference information. However, current studies of early brain development based on cortical morphology mainly focus on statistical analysis of morphological features, while exploration of the SCN is relatively limited, especially at the individual level. The neuroimaging research for early brain development based on SCN is still in its infancy, more work needs to be done to better explore the neural mechanisms of brain development.

This opinion paper aims to comprehensively analyze the relevant literature on early brain development from the perspectives of cortical morphological indicators and SCNs during both the fetal period and the first 5 years of life. We will delineate the research significance of SCNs in the context of early brain development, while also addressing the existing challenges and outlining potential directions for future research. It is expected that these analyses will shed light on the field of early brain development based on morphological SCN.

## 2. Morphological SCN in early brain development

### 2.1. Developmental patterns during the fetal period

The fetal period is a crucial stage in early brain development, marked by the prolific formation of dendrites and synapses within the cortical plate and significant transformations in cerebral cortical morphology (Dubois et al., 2007; Cao et al., 2017). During this phase, the cerebral cortex rapidly develops from an initial smooth structure without gyri and sulci into an extremely complex folded structure (Dubois et al., 2007). With the formation of sulci-gyri, the complexity of the cerebral cortex also increases. This cerebral developmental process unfolds progressively, with primary cortical folding emerging around 20 gestational weeks, followed by the initiation of secondary cortical folding at approximately 32 gestational weeks (Feess-Higgins and Larroche, 1987; Dubois et al., 2019). It has been found that the total brain tissue volume undergoes a nearly 3-fold linear augmentation from 29 to 41 gestational weeks, among which, GMV demonstrates an almost 4-fold increase (Hüppi et al., 1998; Anderson et al., 2015). Concurrently, SA undergoes a substantial expansion, expanding nearly 4-fold between 27 and 40 gestational weeks (Dubois et al., 2019), with the lateral surface generally showing faster growth rates than the ventral and medial surfaces. Furthermore, the developmental pace of SA manifests heterogeneity among distinct cortical regions. Notably, SA of most primary cortices develops faster than the high-order association cortices, such as sensorimotor areas and auditory areas, showing a weekly average increment of more than 25% during 26–29 gestational weeks (Xia et al., 2019).

Currently, research on fetal development using the cortical morphology-based SCN remains relatively understudied. It has been shown that the SCN exhibits dynamic topological reorganization during this period. Notably, due to the challenges associated with obtaining high-quality fetal brain images, premature infants are often utilized as a means to simulate early brain development in the prenatal stage. A study involving premature infants that established the SCN based on SD demonstrated that in premature infants without or with slight injuries, the global efficiency of SCN increases gradually with age during 26.7–43 gestational weeks (age at scan), whereas the characteristic path length decreases with age (Kim et al., 2020). By 40 gestational weeks, premature infants without or with slight injuries exhibited no significant differences in SD and network connection strength compared to full-term infants. These findings suggest that the information integration capacity of the brain network is continuously enhancing during the third trimester.

### 2.2. Developmental patterns during the first 5 years of life

The first 5 years of life is an important period for early postnatal development. At full-term birth, the cortical folding pattern of neonatal brain largely resembles that of adults (Duan et al., 2017b, 2019), and exhibits sex differences, hemispheric asymmetry, and individual differences (Hill et al., 2010; Li et al., 2014a; Duan et al., 2019, 2020). Notably, 0–2 years old is the most dynamic stage of postnatal brain development. Specifically, the brain volume expands rapidly and new tertiary cortical folds appear (Feess-Higgins and Larroche, 1987; Gilmore et al., 2007; Dubois et al., 2019). Various cortical morphological features, e.g., GMV, CT, SA, SD, folding index, and multiscale decompositions of cortical curvature, exhibit dramatic developmental changes during this period. The cortical development rate for most of these features is substantially higher during the first year than in the second year, with growth rates slowing down after 2 years of age (Li et al., 2014b, 2015; Lyall et al., 2015; Duan et al., 2017a, 2020). Specifically, during 0–1 years old, infant GMV increases about 108–149%; during 1–2 years old, GMV increases by about 14–19%; after 2 years old, GMV undergoes minor growth throughout childhood and exhibits a decline during adolescence (Knickmeyer et al., 2008; Groeschel et al., 2010; Gilmore et al., 2011, 2018). Regarding CT of infant brain, there is an average increase of 31% during 0–1 years old, followed by a growth of 4.3% during 1–2 years old, ultimately reaching 97% of adult thickness by 2 years old (Li et al., 2015; Lyall et al., 2015; Gilmore et al., 2018). It is noteworthy that CT peaks at around 14 months, and subsequently undergoes a gradual thinning process during adolescence (Lyall et al., 2015; Wang et al., 2019).

Currently, research on SCN-based early brain development patterns of children across the first 5 years of life is still in a nascent stage. Studies have found that in full-term neonates, SCN already exhibits the small-world topology and ordered modular organization (Fan et al., 2011; Meng et al., 2015). During 0–2 years old, the global efficiency, local efficiency, clustering coefficient, and modularity index of SCN constructed based on CT increases with age, and the shortest path length decreases with age (Fan et al., 2011; Meng et al., 2015), which suggest that both network segregation and integration enhances with age. However, a study on infants aged 0–2 years old found that in SCNs constructed based on CT, the global efficiency increases while the local efficiency decreases with age; in SCNs constructed based on curvedness, the global efficiency decreases while the local efficiency increases with age (Nie et al., 2014). Another study on 3–5-year old infants showed that the global efficiency of SCN constructed based on CT and curvedness decreases to varying degrees, while local efficiency increases to varying degrees (Nie et al., 2013). The inconsistencies among these findings may arise from variations in the morphological features and analysis methodologies used for network construction. In addition, a cross-sectional study of infants at 37–44 gestational weeks found age-related associations in the average structural connectivity strength (SCS) of network nodes. Among them, the SCS of sensorimotor cortex, auditory cortex, and visual cortex increases significantly with age (Fenchel et al., 2020; Galdi et al., 2020), while the SCS of cingulate gyrus and limbic cortex declines with age. These findings collectively indicate that after birth, the information integration and transmission capacity of the infant brain networks are significantly enhanced, leading to a more efficient organization.

### 2.3. The research value of morphological SCN in early brain development

Morphological SCNs provide valuable insights into the complex organization of the human brain. These networks reveal organized co-variation of cortical regions' morphological properties, to some extent resembling white matter and functional networks, and have been reported to be associated with specific cognitive and behavioral functions (Chen et al., 2011; Gong et al., 2012; Alexander-Bloch et al., 2013; Seidlitz et al., 2018; Fenchel et al., 2020). In addition, SCNs exhibit genetic heritability and undergo changes across the lifespan, with structure alterations observed in neuropsychiatric disorders, such as Alzheimer's disease and schizophrenia (He et al., 2008; Seeley et al., 2009; Yao et al., 2010). Individual-level morphological networks offer a novel perspective on understanding brain development and neuropsychiatric conditions (Cai et al., 2023). The individual SCNs assess inter-regional morphological similarity in single subjects mainly by estimating the similarity/divergence of regional feature distributions (Li et al., 2021; Sebenius et al., 2023) or by evaluating the correlation of regional feature vectors (Li et al., 2017; Seidlitz et al., 2018). This approach enables the investigation of individual topological brain changes in both healthy and diseased states. The relationship between morphological similarity and gene co-expression enriches our understanding of brain organization. Consequently, SCNs hold substantial neuroscientific significance in studying early brain development, contributing to the comprehension of cognitive development and risk for neuropsychiatric disorders in early childhood.

### 2.4. Current challenges

Despite pioneer studies on early brain development from the perspective of SCN, there are still many challenges to overcome. Notably, due to the considerable challenges in image acquisition and processing for fetal and infant populations, the corresponding datasets are scarce compared with adults, which also leads to the current research remaining in a nascent stage. Besides, most present studies are based on group-level SCN, which could not describe the individual information for further deep analysis of individual development. Compounding this, the development patterns from diverse studies are not very consistent, attributable to the heterogeneity in SCN construction methodologies and distinct morphological features employed (Alexander-Bloch et al., 2013; Nie et al., 2014). Furthermore, the relationship between cortical morphological development and cognitive function development is still unknown. More work needs to be done to further understand the early brain development.

### 2.5. Future direction of morphological SCN in early brain development

Morphological SCNs hold great potential for exploring the neural mechanism of early brain development as well as neurodevelopment disorders. Many directions in the future require more attention. For example, (1) Propose better segmentation and registration methods to accurately analyze the fetal images with low SNR to construct SCNs to reveal the network properties, such as small-worldness, modularity, and hub distributions, during the fetal stage. (2) Appropriately combine the worldwide human brain development datasets, such as the Developing Human Connectome Project (dHCP, http://www.developingconnectome.org/project/), Baby Connectome Project (BCP, https://babyconnectomeproject.org/), Adolescent Brain Cognitive Development (ABCD, https://abcdstudy.org/) study, and upcoming datasets from the China Brain Project, to comprehensively explore the brain development patterns over a wide range of age durations. (3) Evaluate the influences of various factors and analysis methodologies in network construction, and explore reliable early brain developmental patterns. (4) Apply new individual-level SCN methods (Li et al., 2021; Sebenius et al., 2023) to early brain development studies that estimate the structural connectivity between cortical regions based on the similarity of feature distributions, thereby further analyzing the individual brain development of brain maturation. (5) Explore the relationships of developmental patterns among SCNs, functional networks, and white matter connectivity networks, as well as the relationships between the development of individual SCNs and cognitive functions. These studies will benefit the understanding of the neural mechanism of early brain development in both healthy individuals and individuals with neurodevelopment disorders; and combined with the brain-computer interface (BCI) control technology, which may contribute to the evaluation and rehabilitation of children with conditions such as developmental delays and movement disorders.

## 3. Conclusions

In conclusion, this study reviewed the recent literature on early brain development based on morphological SCNs. Specifically, the morphology of cerebral cortex develops dramatically during the first few years of life, with various morphological features, e.g., GMV, CT, and SA, significantly multiplying in this period. In addition, the SCN of full-term newborns exhibits small-world topology and ordered modular organization, showing dynamic changes in network separation and integration, suggesting that the brain is moving toward an organization with higher information integration and transmission capacity during early brain development. However, this field is still in a nascent stage, more work needs to be done. Our opinion is that it is necessary to apply new individual-level SCNs instead of traditional group-level SCNs and utilize databases spanning multiple age groups, to facilitate extensive analyses on a large sample level to explore early brain development patterns and individual differences; to reveal the relationship between developmental patterns among SCN, functional networks, and white matter connectivity networks; and to investigate the relationship between individual morphological development and cognition development. These studies based on SCN will deepen our understanding of the neural mechanism underlying early brain development as well as neurodevelopment disorders. This will contribute to optimizing the design of effective BCI systems, which could ultimately provide substantial help for children with neurodevelopmental disorders, e.g., developmental delays and movement disorders, to restart normal life in the near future.

## Author contributions

DD: Writing—original draft, Writing—review & editing. DW: Writing—review & editing, Supervision.
